# Validation of the Persian version of the Perinatal Anxiety Screening Scale (PASS) among antenatal and postnatal women

**DOI:** 10.1186/s12884-022-05217-6

**Published:** 2022-11-29

**Authors:** Parastoo Amiri, Kambiz Bahaadinbeigy, Fatemeh Asadi, Shoboo Rahmati, Shahrzad Mazhari

**Affiliations:** 1 Student Research Committee, Kerman University of Medical Sciences, Kerman, Iran; 2 Medical Informatics Research Center, Institute of Futures Studies in Health, Kerman University of Medical Sciences, Kerman, Iran; 3 Health Information Technology, School of Management and Medical Information, Kerman University of Medical Sciences, Kerman, Iran; 4 Neuroscience Research Center, Institute of Neuropharmachology, Kerman University of Medical Sciences, Kerman, Iran

**Keywords:** Validation, Reliability, Pregnancy, Anxiety, PASS, Perinatal, Antenatal, Postnatal, Iran

## Abstract

**Background:**

Anxiety disorder is more common in women than men. To some extent, it can be attributed to childbirth and factors related to pregnancy in women. Therefore, it is necessary for mothers to use valid and reliable scale to assess perinatal anxiety, such as the perinatal anxiety screening scale (PASS). The purpose of this study was to investigate the validity and reliability of the PASS in Persian language.

**Methods:**

The PASS was translated into Persian (PASS-IR). Generally, 224 women antenatal and 125 postnatal answered the questions of PASS, EPDS-10, BAI and DASS-21 questionnaires. The data was collected in the health centers of Kerman by random sampling method. Finally, content validity, factor analysis, internal consistency and test-retest reliability were evaluated.

**Results:**

The mean age of the participants was 32.89 years (range between 18 and 45 and SD = 6.23). More than half of the participating were at risk of severe anxiety (53.5%). Content Validity Index (CVI) and Content Validity Ratio (CVR) were 0.80 and 0.87. PASS-IR subscales include social anxiety and specific fears, general anxiety and adjustment, acute anxiety and trauma, and perfectionism and control. PASS-IR was significantly correlated with EPDS-10 (rho = 0.42), BAI (rho = 0.53), DASS-21 with three concepts of depression, anxiety and stress (rho = 0.51, rho = 0.49 and rho = 0.49), and adverse life events (rho = 0.30).

**Conclusion:**

The results of this study show that PASS-IR has good validity and reliability. Therefore, it can be used to screen for anxiety disorder among Iranian women in the perinatal stage.

## Introduction

One of the integral parts of human life in facing danger and threatening situations is anxiety and fear [1]. There are different types of anxiety disorders, each of which has its own characteristics. In the last two decades, anxiety disorders have been considered the most common mental disorders in the world [2–4]. According to a report from the World Health Organization (WHO), 1 in every 8 people suffered from mental disorders in 2019 [5]. During the Covid-19 pandemic, WHO reported that the prevalence of anxiety and depression in the world has increased by 25% [6]. Of course, in general, the prevalence of anxiety disorders in developed countries is higher than in developing countries [7]. Statistics show that the prevalence of anxiety disorders is high in Iran (15.6%). This disease is more common in women (19.4%) than men (12%) [8]. The reasons for this difference have been discussed in several studies such as the possible role of genetics in combination with environmental factors [9], the possible role of female sex hormones and related cycles [10, 11] and etc. In addition to the stated reasons, part of this anxiety and fear in women is related to their pregnancy and motherhood, which is called pregnancy anxiety [12, 13].

Pregnancy anxiety often refers to concerns about miscarriage, fetal health, childbirth, postpartum care, and financial issues [14–16]. For the first time, the understanding of perinatal anxiety was proposed in the early 20th century by Pleshette et al. [17]. The perinatal anxiety is common in pregnant mothers [18]. In some studies, the prevalence of anxiety during pregnancy has been reported as 20% [19, 20]. Also, several studies have shown that almost one third of pregnant women may experience anxiety symptoms during pregnancy [12, 21, 22]. Untreated anxiety during pregnancy may affect biological indicators such as the height, weight and head circumference of the baby, psychiatric disorders in adulthood such as schizophrenia and mood disorders [23–26]. It can also lead to autism spectrum disorders, hyperactivity, sleep disorders and behavioral problems in early childhood [27–29]. As prenatal anxiety can have long-lasting effects on child and adult psychopathology [30]. This change in the mother’s mood may affect the brain development of the fetus and ultimately affect the child’s behavioral development [24]. A study in 2019 showed that psychiatric interventions can improves the mental health of pregnant mothers [31].

Screening tools are needed for early identification of problematic anxiety, then psychiatric interventions can be delivered to women who need them [32–34]. To do this, we need a scale that, while having sufficient and necessary validity and reliability, can adapt to the perinatal period and be clinically useful [35]. So far, several questionnaires have been designed and validated for this purpose [34, 36]. Somerville et all. [37] found the perinatal anxiety screening scale (PASS) useful for determining the intensity of perinatal anxiety. In Turkey, the Turkish version of this scale (PASS-TR) had high sensitivity and specificity with a cutoff value of 16 [38]. Also, PASS-TR had a high total internal consistency and a positive correlation with other valid and reliable measures of anxiety. In Saudi Arabia, the content validity index (CVI) and content validity ratio (CVR) for the PASS were 0.88 and 0.79, respectively [39]. A study in Italy showed that the PASS identified 98% of Italian mothers with the diagnosis of anxiety disorder [40]. In South America, Spanish version of the PASS developed had good psychometric properties and adequate validity [41]. In Iran, this scale has been validated in Ardabil city with acceptable CVI (0.8-1) and CVR (0.6-1) [42]. But the native language of people in Ardabil city is Turkish, while this scale was translated into Persian. Also, in their study, they included only antenatal women. While this scale examines antenatal and postnatal women. For this purpose, we decided to validate PASS in order to increase its usefulness as a screening tool to identify anxiety during the antenatal and postnatal periods in a population whose native language is Persian.

## Method

### Participants characteristics and data collection

This is a cross-sectional study (descriptive-analytical) and a type of psychometric study. The data were collected from March 2021 to June 2021 and from the health centers of Kerman city (the largest province in Iran and located in the south of it) with different economic and social fields. With the cluster sampling method, out of 21 active health centers in Kerman city, seven centers that covered the largest number of pregnant women were selected. The required sample size for each centre was determined using the proportional to size method. Women were randomly selected upon entering the health centers. In general, 314 women (224 antenatal and 125 postnatal) were included in the study in order to measure the validity and reliability of PASS-IR (Persian version of PASS). According to Osborne and Costello study [43], the sample size was sufficient. Also, the sample size is determined according to the number of scale items.

After explaining the objectives of the research and the confidentiality of their information, the researcher invited the selected mothers to cooperate. Mothers were eligible for participation in the current study if they were at least 18 years old, and if they could read and speak Persian who gave birth during one season (three months) or pregnant women who went these centers for follow-up in the third trimester of pregnancy. Questionnaires were randomly distributed and collected by the first researcher in selected health centers among the participants. Before completing the questionnaire, written consent was obtained from each participant. Completing each questionnaire took an average of 25 min.

### Ethical considerations

Before starting the study, the necessary permissions were obtained from the Ethics Committee of Kerman University of Medical Sciences (KUMS) (code: IR.KMU.REC.1399.189) to conduct the research. The participants were assured of their information confidentiality. Moreover, after explaining the study objectives and the voluntariness of their participation, the participants gave their consent to participate in the study.

### Translation process and validation

Considering that the PASS was developed for English-speaking people, for this purpose, after correspondence with the designer of the PASS and obtaining her permission, the process of translating this scale was done from English to classical Persian using the Forward-Backward method. The PASS was first translated by two independent professional translators who are proficient in both English and Farsi languages and after combining the initial translations into a single translation, the it was translated from Persian to English by two other independent professional translators. Then, in order to ensure the correctness of transferring the concepts, the English translation was matched with the original text of the PASS by the main author of the questionnaire (Somerville et al.). In order to reliability the content of the translated Farsi version, the PASS-TR was approved by 5 gynecologists and obstetricians so that each of the items of the scale can be evaluated in terms of relevance and cultural appropriateness by them. For the purpose of formal reliability, the translated Persian version was given to 20 perinatal women to ensure the clarity of all items. In addition, the convergent validity of PASS was evaluated for the participants of the study by simultaneously implementing the Farsi versions of “Beck Anxiety Inventory (BAI)”, “Depression, Anxiety and Stress Scale (DASS-21)” and “Edinburgh Postpartum Depression Scale (EPDS-10)”. It is necessary to know that the Persian version of these comparisons are valid and reliable [44–46].

In addition to this, demographic characteristics and other information were asked at the beginning of the questionnaire, such as age, level of education (academic, non- academic), marital status (married, divorced/separated/widowed), occupation (house wife, employee), wife’s occupation (if married) (employee, unemployed), total income (< 70000000, **≥** 70000000), pregnancy stage (antenatal, postnatal), gravity (nully, multi), pregnancy (wanted, unwanted, non programed), and anxiety level (low, moderate, severe).

Since negative life events during pregnancy have unpleasant consequences for mothers and later for children [47, 48], we decided to add questions related to adverse events experienced in the past years of life to the questionnaire, such as divorce/separated, problems at work, financial problems, lost someone close, illness in the family, conflict with friends/family, seriously ill/injured, serious accdent/fire/robbery, exposure to any life event during pregnancy, and pregnancy problems. Jradi et al. [39] also reported in their study that there is a positive correlation between adverse life events and PASS, and measuring this adds to the validity of PASS.

### Research tools

#### Perinatal Anxiety Screening Scale (PASS)

In this study, information was collected through PASS. This questionnaire examines the anxiety symptoms of child-bearing women in the last month. The PASS was created by Somerville et al. [37]. This questionnaire has 31 items that have 4 options for each question. Scoring for each item is from 0 to 3. Choosing the option never has a score of “0”, sometimes “1”, often “2” and always “3”. A high score is a sign of more perinatal anxiety. The total score may range from 0 to 93. Cutoff for clinical anxiety is > = 26. In a study, Somerville et al. [49] classified the anxiety of pregnant women into three categories: mild (0–20), mild to moderate (21-41) and severe (42–93).

#### Edinburgh Postpartum Depression Scale (EPDS-10)

This scale has 10 items and is made in such a way that it allows the diagnosis of depression up to six weeks after childbirth [50]. The score of the Edinburgh scale is between 0 and 30, and a score of at least 12 is considered as postnatal depression.

#### Beck Anxiety Inventory (BAI)

BAI is designed to measure the level of anxiety. It consists of 21 statements, for each statement there are four options to choose from zero to three. The total score ranges from 0 to 63. Each phrase reflects one of the symptoms of anxiety that are usually experienced by people who are clinically anxious or who are in a state of anxiety. Finally, the anxiety score of this questionnaire is classified into four categories: minimal (0–7), mild (8-15), moderate (16-25) and severe (26-63) [51, 52].

#### Depression, Anxiety and Stress Scale (DASS-21)

This questionnaire was created by Lavibond and has two forms [53]. Its main form has 42 statements that evaluate each of the mental constructs of depression, anxiety and stress by 14 different statements. Its short form contains 21 statements that measure each of the psychological constructs of depression, anxiety and stress by 7 different statements. The range of questionnaire scores is presented in Table 1.

**Table 1:** Classification of DASS-21 scores

	**Depression**	**Anxiety**	**Stress**
Normal	0–9	0–7	0–14
Mild	10–13	8–9	15–18
Moderate	14–20	10–14	19–25
Severe	21–27	15–19	26–33
Extremely Severe	> = 28	> = 20	> = 34

### Statistical analysis

The responses of the participants were collected anonymously through paper questionnaires. Then the data were entered into SPSS 16 software for statistical analysis. The scoring of all the scales of this research was based on the recommendations. Mean and standard deviation were calculated for each scale. Composite variables were generated based on the final scores as high, moderate, or low to indicate levels of psychological distress among participants for all scales. Study variables were summarized, in aggregate, using standard descriptive statistics and frequencies of responses were reported. Content validity Ratio and Content Validity Index were calculated for content validity check. Factor structure of the questionnaire was performed using principal component analysis (PCA) with oblimin rotation. The number of factors was determined by Velicer´s MAP test. The reliability of the scale was checked via internal consistency and test-retest reliability. Cronbach’s alpha coefficient was reported for internal consistency (alpha ≥ 0.70 was considered acceptable) [54]. Test-retest reliability was conducted (*N* = 20) within one week interval and a correlation coefficient was obtained.

## Results

### Participants characteristics

In general, 354 women were selected for this study. Finally, 314 people were included in the study because 40 of them were not included in the final sample size (20 people were considered for the initial pilot stage and 20 people were considered for test-retest reliability). The characteristics of the participants are presented in Table 2. Their average age was 32.89 years (range between 18 and 45) with a standard deviation of 6.23 years. More than half of the participants had a university degree (53.82%). Almost half of them were employed (45.54%). Since the poverty line in Iran is approximately 70,000,000 Rials per month (219 $), the family income of more than half of them was not enough for a comfortable life (56.36%). Most perinatal women were multi gravity (63.71%) and about half of postnatal women were nully (49.34%). Most of the multi gravity women got pregnant unwanted or non programed (74.03%). In the English version of the PASS scale, the cut-off values ​​are 0–20: minimal anxiety, 21–41: moderate anxiety, and 41 or higher reports severe anxiety [37]. Based on this, in the present study, almost half of participants were at risk of severe anxiety (53.50%).

**Table 2:** Characteristics of participants

**Characteristics**		**NO. (%)**
Age (Year)	18–25	66 (21.02)
	26–35	131 (41.72)
	≥ 36	117 (37.26)
	Mean ± SD	32.89 ± 6.23
Level of Education	University degree	169 (53.82)
	Complete high school	78 (24.84)
	Incomplete high school	49 (15.61)
	Incomplete university degree	18 (5.73)
Marital Status	Married	298 (94.90)
	Divorced/Separated/Widowed	16 (5.10)
Occupation	House wife	171 (54.46)
	Employee	143 (45.54)
Wife’s occupation	Employee	186 (59.24)
	Unemployed	128 (40.76)
Total income (IR)^a^	< 70000000	177 (56.36)
	≥ 70000000	137 (43.63)
Pregnancy Stage	Antenatal	200 (63.69)
	Postnatal	114 (36.30)
Gravity	Nully	116 (36.94)
	Multi	198 (63.06)
Pregnancy	Wanted	254 (80.89)
	Unwanted	23 (7.32)
	Non programed	37 (11.78)
Level of Anxiety (PASS)	Low	57 (18.15)
	Moderate	89 (28.34)
	High	168 (53.50)

^a^IR Iran Riyals

Table 3 shows the frequency of answers to each of the questions of the PASS-IR. In Table 4, the mean score of the Persian-IR was 45.63 with a standard deviation of 14.42 (range: 0 to 93). The mean EPDS-10 score was 19.65 (SD = 4.01) (range 0 to 30). 72.15% of participating women were at risk of depression (EPDS-10 score was more than 13) [50]. The average score of the BAI was 27.41 (SD = 6.32), which indicated the level of severe anxiety in the participating mothers. Also, based on the DASS-21 scale, 42% of participating mothers were at risk of severe and very severe anxiety. The mean scores of the three DASS-21 components were 13.04 (SD = 8.50) for depression, 13.23 (SD = 9.56) for anxiety, and 19.10 (SD = 11.24) for stress symptoms.

**Table 3:** Participants’ responses to the PASS-IR (*N* = 314)

NO.	**Questions**	**Not at all N(%)**	**Sometimes N(%)**	**Often N(%)**	**Almost Always N(%)**
1	Concern about pregnancy or baby	44 (12.05)	200 (57.00)	74 (21.01)	27 (7.07)
2	Fear that the baby will be harmed	39 (11.10)	193 (55.00)	80 (22.08)	39 (11.10)
3	Feeling of intense fear that something bad will happen	103 (29.30)	181 (51.60)	40 (11.40)	24 (6.80)
4	Worry about many things	61 (17.40)	210 (59.80)	55 (15.70)	17 (4.80)
5	Concern about the future	107 (30.50)	128 (36.50)	67 (19.10)	43 (12.30)
6	Feeling overwhelmed	220 (62.70)	80 (22.80)	26 (7.40)	15 (4.30)
7	Very intense fear about some things like: needles, blood, childbirth, pain and …	133 (37.90)	105 (29.90)	67 (19.10)	43 (12.30)
8	Sudden feeling of fear or discomfort	111 (31.60)	156 (44.40)	61 (17.40)	18 (5.10)
9	Repetitive thoughts that are difficult to stop or control	130 (37.00)	152 (43.30)	53 (15.10)	11 (3.10)
10	Difficulty sleeping even when I have enough time to sleep	112 (31.90)	137 (39.00)	66 (18.80)	31 (8.80)
11	Insist on doing things in a certain way or order	98 (27.90)	120 (34.20)	66 (18.80)	62 (17.70)
12	The desire for everything to be perfect	40 (11.40)	88 (25.10)	89 (25.40)	134 (38.20)
13	Need to control everything precisely	57(16.20)	142 (40.50)	70 (19.90)	51 (14.50)
14	Difficult to stop my repeating tasks or checks	108 (30.80)	151 (43.00)	53 (15.10)	21 (6.00)
15	Sudden feeling of jerking and cringing	152 (43.30)	150 (42.70)	41 (11.70)	8 (2.30)
16	Worry about repeating thoughts	99 (28.20)	169 (48.10)	49 (14.00)	31 (8.80)
17	Being on guard or needing to do watch out for things	162 (46.20)	105 (29.90)	51 (14.50)	30 (8.50)
18	Worry about recurring memories, dreams or nightmares	168 (47.90)	116 (33.00)	31 (8.80)	28 (8.00)
19	Worrying about being embarrassed in front of other peoples	232 (66.10)	82 (23.40)	13 (3.70)	16 (4.60)
20	Fear of being judged negatively by others	183 (52.10)	106 (30.20)	32 (9.10)	27 (7.70)
21	Feeling deep discomfort in crowded places	214 (61.00)	103 (29.30)	24 (6.80)	10 (2.80)
22	Avoid social activities because I may get nervous	210 (59.80)	80 (22.80)	42 (12.00)	15 (4.30)
23	Stay away from the things that worry me	94 (26.80)	185 (52.70)	57 (16.20)	13 (3.70)
24	Feeling unreal, I feel like I'm watching a movie about myself	247 (70.40)	65 (18.50)	22 (6.30)	7 (2.00)
25	Loss of time and inability to remember what happened	163 (46.40)	106 (30.20)	38 (10.80)	27 (7.70)
26	Failure to adapt to recent changes	172 (49.00)	130 (37.00)	30 (8.50)	17 (4.80)
27	Anxiety can impair the ability to do things	98 (27.90)	167 (47.60)	41 (11.70)	45 (12.80)
28	The influx of thoughts makes it difficult to concentrate	129 (36.80)	151 (43.00)	51 (14.50)	14 (4.00)
29	Fear of losing Control	182 (51.90)	111 (31.60)	36 (10.30)	18 (5.10)
30	Sudden feeling of fear and anxiety	193 (55.00)	116 (33.00)	19 (5.40)	18 (5.10)
31	Feeling of restlessness and confusion	123 (35.00)	154 (43.90)	38 (10.80)	32 (9.10)

**Table 4:** Distributions of scales (medians, means, and standard deviations) and correlations with PASS

**Scale**	**Median**	**Mean (SD)**	**Range**	**Spearman’s Rho (** ***p-*** **value)**
PASS-IR	41	45.63 (14.42)	3–97	-
EPDS-10	20	19.65 (4.01)	6–26	0.42 (< 0.001)
BAI-21	23	27.41 (6.32)	5–63	0.53 (< 0.001)
DASS-21 (depression)	10	13.04 (8.50)	0–42	0.51 (< 0.001)
DASS-21 (anxiety)	12	13.23 (9.56)	0–42	0.49 (< 0.001)
DASS-21 (stress)	18	19.10 (11.24)	0–42	0.49 (< 0.001)
Adverse life events				
Divorce/Separated	0	0.42 (1.23)	0–3	0.21 (0.10)
Problems at work	0	0.56 (1.62)	0–3	0.11 (0.002)
Financial problems	1	0.23 (0.46)	0–3	0.16 (0.42)
Lost someone close	0	0.39 (0.35)	0–3	0.07 (0.02)
Illness in the family	0	0.85 (1.27)	0–3	0.12 (0.007)
Conflict with friends/family	0	0.97 (1.51)	0–3	0.13 (0.0005)
Seriously ill/injured	0	0.95 (1.17)	0–3	0.22 (0.07)
Serious accident/fire/robbery	0	0.87 (1.12)	0–3	0.12 (0.005)
Exposure to any life event during pregnancy	1	1.55 (1.31)	0–3	0.05 (0.002)
Pregnancy problems	1	1.32 (1.19)	0–3	0.18 (0.07)

### Validity

Convergent validity was used to evaluate the instrument’s validity. Mean Content Validity Ratio (CVR) and mean Content Validity Index (CVI) were 0.80 and 0.87 respectively. Correlation between Persian version of PASS, Persian version of EPDS-10 (Spearman’s rho = 0.42, *p* < 0.001), Persian version of BAI version (Spearman’s rho = 0.53, *p* < 0.001), the three components of DASS-21 (depression, anxiety, and stress) (Spearman’s rho = 0.51, *p* < 0.001; Spearman’s rho = 0.49, *p* < 0.001; Spearman’s rho = 0.49, *p* < 0.001) and the score of adverse life events (Spearman’s rho = 0.30, *p* = 0.003) in the past 3 months were significantly positive. In general, the results show that people who had perinatal anxiety had higher levels of mental disorders and higher levels of depression. Particularly those who reported experiencing divorce/separated, financial problems, seriously ill/injured, and pregnancy problems significantly correlated with the PASS scores (Table 4).

### Reliability

Internal consistency using Cronbach’s alpha coefficient as a measure of reliability of the Persian version of the PASS questionnaire was found to be 0.95 for the whole sample indicating very satisfactory results. It was 0.83, 0.80, 0.86, and 0.90 for the four sections of the scale. The results of the test-retest reliability of the Persian version of the PASS tool showed to be acceptable with a correlation coefficient of 0.76.

### Factor structure

In Table 5, the results for the factor structure of the scale are reported. In order to confirm the structure of the Persian version of the instrument and demonstrate its construct validity and similarity to the English version, the Principal Component Method for factor analysis with varimax rotation was applied using the original four factor structure of the instrument. The four factors jointly accounted for 56.83% of the total detected variance. Factor 1 (social anxiety and specific fears) accounts for 19.16% of the total variance, factor 2 (general anxiety and adjustment) 14.89%, factor 3 (acute anxiety and trauma) 12.74%, and factor 4 (perfectionism and control) 10.05%.

**Table 5:** Factor structures and factor loadings

**PASS Items**	**Factor 1**	**Factor 2**	**Factor 3**	**Factor 4**
**Factor 1: Social anxiety and Specific fears**				
20. Fear of being judged negatively by others	0.79			
19. Worrying about being embarrassed in front of other peoples	0.74			
30. Sudden feeling of fear and anxiety	0.74			
18. Worry about recurring memories, dreams or nightmares	0.70			
31. Feeling of restlessness and confusion	0.65			
22. Avoid social activities because I may get nervous	0.64			
8. Sudden feeling of fear or discomfort	0.60			
6. Feeling overwhelmed	0.57			
28. The influx of thoughts makes it difficult to concentrate	0.54			
21. Feeling deep discomfort in crowded places	0.50			
24. Feeling unreal, I feel like I'm watching a movie about myself	0.50			
23. Stay away from the things that worry me	0.41			
**Factor 2: General anxiety and Adjustment**				
26. Failure to adapt to recent changes		0.82		
16. Worry about repeating thoughts		0.66		
25. Loss of time and inability to remember what happened		0.60		
29. Fear of losing Control		0.58		
27. Anxiety can impair the ability to do things		0.56		
15. Sudden feeling of jerking and cringing		0.48		
17. Being on guard or needing to do watch out for things		0.47		
**Factor 3: Acute anxiety and Trauma**				
3. Feeling of intense fear that something bad will happen			0.82	
2. Fear that the baby will be harmed			0.79	
9. Repetitive thoughts that are difficult to stop or control			0.68	
1. Concern about pregnancy or baby			0.64	
4. Worry about many things			0.62	
5. Concern about the future			0.47	
7. Very intense fear about some things like: needles, blood, childbirth, pain and …			0.42	
**Factor 4: Perfectionism and Control**				
13. Need to control everything precisely				0.79
12. The desire for everything to be perfect				0.77
14. Difficult to stop my repeating tasks or checks				0.65
11. Insist on doing things in a certain way or order				0.63
10. Difficulty sleeping even when I have enough time to sleep				0.39

#### Discussion

Since perinatal anxiety is a common mental health problem for Iranian mothers, we decided to conduct a study with the aim of evaluating and validating the PASS scale to check the power of this test as a perinatal screening in Iran. Different age groups of women with different socioeconomic status participated in this study. Also, mothers participated in two stages three months before and after childbirth. The results showed that the PASS scale is suitable for both stages. In this study, the PASS-IR was conducted on a sample of 314 people and was evaluated with equivalent tests such as EPDS-10, BAI and DASS-21.

The results of the present study showed that some mothers suffer from anxiety disorders. PASS-IR had a positive and significant relationship with previously validated Persian versions of EPDS-10, BAI and DASS-21. Since anxiety and depression occur at the same time more than 50% [55], PASS had a positive correlation with EPDS in this study. Also, PASS had a positive and significant relationship with adverse life events, but this relationship was weak. The reason for this also depends on how people deal with adverse events in their lives [56]. For example, a person may experience very adverse life events but get a low PASS-IR score. This is because of the favorable strategies that person uses to deal with these adverse events. Therefore, more studies are very necessary in relation to adverse events experienced in life and anxiety.

In Western Australia, the original version of the PASS consists of 4 subscales that were compiled by Somervill et al. in 2014 [37]. The original version of the PASS with a cut-off point of 26 includes the subscale “acute anxiety and adjustment disorder” (PASS 1–8) related to measuring the symptoms of anxiety disorders, separation and adjustment problems, the subscale “general worry and specific fears” (PASS 9) 18- related to the measurement of symptoms of general disorder and fear, the “perfectionism control and trauma” subscale (PASS 19–26) related to the measurement of obsessive-compulsive disorders and post-traumatic stress, and finally the “acute anxiety” subscale (PASS 27–31). However, in PASS-IR, the sub-scales were changed using factor load to “social anxiety and specific fears”, “general anxiety and adjustment”, “acute anxiety and trauma”, and “perfectionism and control”. Our findings are consistent with those of the original instrument. Also, these results of factor analysis are similar to those obtained for the Turkish and Arabic version of the scale on factor 4 (perfectionism and control) and partially for the other three factors. We can be said that these changes are a necessary part of linguistic and cultural adaptations, and the scale of results reflects the specifics of Iranian culture. Actually, we hope that the use of PASS-IR can be helpful in removing barriers when assessing mental health problems (such as anxiety, stress and depression) in the perinatal period.

It is necessary to evaluate anxiety disorders in perinatal through valid and reliable measures [57, 58]. Investigations in the present study showed that PASS-IR is a valid and reliable scale for assessing anxiety symptoms in Persian-speaking populations and has acceptable psychometric properties. The reliability coefficient calculated for PASS-IR showed high reliability (0.95). This is consistent with previous studies. For example, the reliability coefficient in the English version of PASS was 0.95, the Turkish version was 0.96, and the Persian version in Ardabil was 0.96 [37, 38, 42]. Also, the test-retest reliability in our study and in the English version was 0.76 and 0.74, respectively.

In the present study, according to the English version of PASS in Australia, PCA identified a four-factor structure. The composition of these four factors is slightly different from Iranian, SriLankan, Turkish and Bangladeshi versions [38, 42, 59, 60]. However, two studies were conducted in Saudi Arabia, one of which reported a four-factor structure and the other a seven-factor structure [39, 61]. This is normal in studies, especially when several years pass from one factor analysis to the next [62, 63]. Another possible reason is that the specific populations have different characteristics in terms of mental health. Also, when items are translated, they may be interpreted slightly differently from the original versions; cultural aspects are probably involved in this as well.

This study has several limitations. The participants in our study were selected from several health centers in Kerman city. For this reason, the sample of this study cannot be considered representative of the entire province of Kerman or the population of Iran. Since official Farsi was used for the translation and validation of this tool, this has made it understandable and useful for all Persian speaking people. Also, we did not evaluate the validity of the PASS scale in the participants in the entire prenatal and postpartum periods, we should limit our conclusions to the last three months before childbirth and three months after childbirth. We suggest that future studies measure the validity of this scale for the entire period. Another limitation of the present study was the lack of sensitivity and specificity for calculating the cut-off point. In this study, according to the original version, a cut-off point of 26 was considered for the screening of anxiety disorders in the perinatal period. Also, sample size was very small (*N* = 20). This was as a limitation of the study.

## Conclusion

Because the assessment of perinatal anxiety with special scales is very important for the mental health of mothers and upbringing babies. Therefore, in this study, an article with a Persian scale was presented to assess anxiety during antenatal and postnatal periods. PASS-IR is more valid and reliable than other available tools and can be used as a screening tool for anxiety among the Persian speaking population. Our suggestion is that the use of this questionnaire should be included in standard clinical practice. Also, due to the future impact of maternal pregnancy anxiety on child development and parenting style, it is suggested that more studies be conducted in order to reduce the effects of anxiety during pregnancy. In addition, we suggest that a confirmatory factor analysis be conducted in the future to confirm the factor structure found in PASS-IR.

## Data Availability

Our data or material may be available from the first or corresponding author upon reasonable request.
